# Efficacy of blinatumomab as maintenance therapy for B-lineage acute lymphoblastic leukemia/lymphoma following allogeneic hematopoietic cell transplantation

**DOI:** 10.1038/s41408-024-01092-w

**Published:** 2024-07-08

**Authors:** Jiayu Huang, Bingyang Shi, Suhui Yu, Mengxing Xue, Ling Wang, Jieling Jiang, Jiong Hu, Jun Zhu, Suning Chen, Lijing Shen, Weijie Cao, Yang Cao, Xiaoxia Hu

**Affiliations:** 1 State Key Laboratory of Medical Genomics, Shanghai Institute of Hematology, National Research Center for Translational Medicine at Shanghai, Ruijin Hospital, Shanghai Jiao Tong University School of Medicine, Shanghai, 200025 China; 2 Collaborative Innovation Center of Hematology, Shanghai Jiao Tong University School of Medicine, Shanghai, 200025 China; 3 Department of Hematology, The First Affiliated Hospital of Zhengzhou University, Zhengzhou, 450052 Henan China; 4 Department of Hematology, Renji Hospital, Shanghai Jiao Tong University School of Medicine, Shanghai, 200136 China; 5 National Clinical Research Center for Hematologic Diseases, Jiangsu Institute of Hematology, The First Affiliated Hospital of Soochow University, Institute of Blood and Marrow Transplantation, Collaborative Innovation Center of Hematology, Soochow University, Suzhou, 215006 China; 6 Department of Hematology, Shanghai Institute of Hematology, Blood and Marrow Transplantation Center, Ruijin Hospital, Shanghai Jiao Tong University School of Medicine, Shanghai, 200025 China; 7 GoBroad Medical Institute of Hematology (Shanghai Center), Liquan Hospital, Shanghai, 200433 China; 8 Department of Hematology, Tongji Hospital, Tongji Medical College, Huazhong University of Science and Technology, Wuhan, 430030 China

**Keywords:** Acute lymphocytic leukaemia, Haematopoietic stem cells

**TO THE EDITOR:**

Allogeneic hematopoietic stem cell transplantation (allo-HSCT) remains a curative therapy for patients with high-risk B-cell acute lymphoblastic leukemia/lymphoma (B-ALL/LBL) [1]. However, according to the EBMT database, the relapse rate of allo-HSCT recipients during their first complete remission (CR) can reach 22% [2], and post allo-HSCT relapse is associated with a disappointing remission rate and dismal outcomes [3, 4]. Therefore, it is crucial to implement strategies to mitigate the risk of relapse after allo-HSCT. Tyrosine kinase inhibitors are widely used as effective maintenance treatments for Philadelphia (Ph)-positive B-ALL [1, 5]. However, there are few reports on suitable maintenance therapies for Ph-negative B-ALL following allo-HSCT. A single-center retrospective study suggested those with Ph-negative B-ALL may benefit from decitabine maintenance following allo-HSCT, as decitabine more than halved the 3-year relapse rate (19.5% *vs*. 42.2%, *P* = 0.068) [6]. Prophylactic donor-derived CD19 CAR-T cell infusion after allo-HSCT was reported to significantly lower the 2-year cumulative incidence of relapse in high-risk B-ALL to 5.6% [7]. Recently, a phase 1 clinical trial was conducted to investigate the safety and efficacy of low-dose inotuzumab ozogamicin (INO) as a posttransplant maintenance [8]. INO demonstrated a favorable safety profile and 1-year progression-free survival (PFS) of 89%.

Blinatumomab is a bispecific T-cell engager (BITE) molecule that directs CD3^+^ T cells to engage and lyse target CD19^+^ cells [9]. The drug was initially approved for relapsed/refractory (R/R) Ph-negative B-cell precursor (BCP)-ALL based on phase 3 of the TOWER study, and was demonstrated to result in a higher overall response rate and longer median overall survival (OS) compared to standard care. Blinatumomab was further approved for patients with BCP-ALL with persistent or reappearing measurable residual disease (MRD) following phase 2 of the BLAST study [10, 11], and several publications have reported the efficacy of blinatumomab as a salvage therapy for B-ALL patients after allo-HSCT [12]. However, there are few reports describing the efficacy and safety of blinatumomab as a maintenance strategy following allo-HSCT. Furthermore, the potential impact of blinatumomab on transplantation-related complications, such as acute graft-versus-host disease (aGvHD), has not been thoroughly documented. In this real-world study, we aimed to describe the feasibility and clinical benefits of blinatumomab maintenance in high-risk B-ALL/LBL patients after allo-HSCT.

This retrospective, multicenter study was designed based on the transplant databases of Shanghai Ruijin Hospital, Wuhan Tongji Hospital, the First Affiliated Hospital of Soochow University, Shanghai Renji Hospital, Shanghai Zhaxin Hospital, and the First Affiliated Hospital of Zhengzhou University. B-ALL/LBL patients transplanted between January 2022 and October 2023 were screened using the following eligibility criteria: those who (1) had a high risk of relapse following allo-HSCT; (2) achieved CR with undetectable MRD post-HSCT, as assessed by flow cytometry with a sensitivity of 0.01% within the first three months, and received at least one cycle (at least consecutive 7 days) of blinatumomab maintenance; (3) had B-ALL/LBL with CD19 expression; (4) had complete medical information. The last follow-up was on March 10, 2024. All procedures used in this study were in accordance with Declaration of *Helsinki* and were approved by the institutional review board [Ethical approval number: TJ-IRB202402092]. Written informed consent was waived due to the retrospective nature of the study.

The protocols used for the preconditioning regimen, GvHD prophylaxis, infection prophylaxis, and MRD methodology and detection frequency are described in the Supplementary Methods. Data collection ended at the time of death or last follow-up. The primary endpoint was relapse rate. Secondary endpoints included OS, event-free survival (EFS), GvHD-free and relapse-free survival (GRFS), non-relapse mortality (NRM), and adverse events (AEs). All endpoints were measured from the date of transplantation. Treatment interruption was defined as a period exceeding 8 days without the treatment being administered. Statistical analyses were performed using R 4.2.0 software. Continuous and categorical variables were presented as medians and range variables, and counts and percentages, respectively. The Kaplan–Meier method was used to estimate survival probabilities, while the Fine–Gray model was used to calculate the cumulative incidences of relapse rate and NRM. The 95% confidential intervals were calculated, and a *p-*value of < 0.05 was considered statistically significant.

Twenty-one B-ALL patients who received at least one cycle of blinatumomab therapy after allo-HSCT were included in this study. Of the 21 patients, one patient was diagnosed with Ph-positive B-ALL (P16), and the other 20 patients had Ph-negative B-ALL/LBL (Table 1). Supplementary Table 1 summarizes the baseline characteristics of the study cohort. Nine patients (42.9%) were diagnosed as having refractory/relapsed B-ALL/LBL. Within the B-ALL group, one patient had extramedullary involvement at diagnosis (P14, central nervous system) that resolved before transplantation, and one patient had extramedullary relapse during treatment and did not go into remission prior to transplantation (P17, central nervous system and testicles). Patient treatment histories before allo-HSCT are detailed in Supplementary Table 2.

**Table 1:** Detailed information of recipients receiving blinatumomab.

Patient	Age	Sex	Primary diseases					Allo-HSCT profile			
			Diagnosis	Molecular alterations	R-DRI	Cytogenetic and molecular prognostic risk^a^	Refractory/relapsedB-LBL/ALL	Disease status pre-HSCT	Molecular alterations pre-HSCT	Donor Type	Days from diagnosis to HSCT
P1	59	Male	Ph-negative B-ALL	*PAX5::ESRRA*, *FLT3-TKD, NRAS, PAX5, KRAS, KDM6A*	Intermediate risk	High risk	N	CR1 with undetectable MRD	Negative	MUD	172
P2	17	Male	Ph-negative B-ALL	*EP300::ZNF384, EP300*	Intermediate risk	High risk	N	CR1 with undetectable MRD	Negative	HID	180
P3	60	Male	Ph-negative B-ALL (secondary to AA)	*IDH1, IKZF1*	Intermediate risk	High risk	Y	CR1 with undetectable MRD	Negative	HID	217
P4	47	Female	Ph-negative B-ALL	*CREBBP::ZNF384, KRAS*	Intermediate risk	High risk	N	CR1 with undetectable MRD	Negative	HID	173
P5	46	Male	Ph-negative B-ALL	*MEF2D::HNRNPUL1*	Intermediate risk	High risk	N	CR1 with undetectable MRD	NA	HID	382
P6	50	Female	Ph-negative B-ALL	*NRAS, FLT3-ITD, ASXL1, CDKN2B, SYNRG::ZNF384*	Intermediate risk	High risk	N	CR1 with undetectable MRD	Negative	HID	162
P7	27	Female	Ph-negative B-ALL	*IKZF1 deletion, CDKN2A, EPOR::IGH*	Intermediate risk	High risk	N	CR1 with undetectable MRD	Negative	HID	204
P8	33	Male	Ph-negative B-ALL	*KMT2A::AFF1*	Intermediate risk	High risk	N	CR1 with undetectable MRD	Positive	HID	162
P9	48	Female	Ph-negative B-ALL	*KMT2A::AFF1*	Intermediate risk	High risk	N	CR1 with undetectable MRD	Negative	HID	96
P10	50	Male	Ph-negative B-ALL	*KMT2A::AFF1*	Intermediate risk	High risk	N	CR1 with undetectable MRD	Positive	HID	119
P11	22	Male	Ph-negative B-ALL	*IgH rearrangement*	High risk	High risk	Y	CR2 with undetectable MRD	Negative	HID	2310
P12	29	Female	Ph-negative B-ALL	*EP300::ZNF384, NRAS*	High risk	High risk	Y	CR2 with undetectable MRD	Negative	HID	4768
P13	14	Male	Ph-negative B-ALL	*IgH rearrangement*	High risk	High risk	Y	CR2 with detectable MRD	Positive	HID	477
P14	19	Male	Ph-negative B-ALL	*CDKN2A deletion, CDKN2B deletion, KRAS, NRAS*	High risk	Standard risk	Y	CR2 with detectable MRD	NA	HID	192
P15	29	Female	B-LBL	*TCF3::PBX1*	High risk	Standard risk	Y	NR	Positive	HID	322
P16	50	Female	Ph-positive B-ALL (CML blast phase)	*BCR::ABL1,IKZF1*	Intermediate risk	High risk	Y	CR1 with undetectable MRD	Negative	HID	237
P17	15	Male	Ph-negative B-ALL	NA	High risk	Standard risk	Y	NR	NA	HID	4752
P18	28	Male	Ph-negative B-ALL	*CRLF2, PAX5, DDX41, EP300*	Intermediate risk	High risk	N	CR1 with undetectable MRD	NA	HID	312
P19	46	Male	Ph-negative B-ALL (secondary to AA)	Negative	Intermediate risk	Standard risk	Y	CR1 with undetectable MRD	NA	MSD	103
P20	19	Male	B-LBL	*IKZF1*	Intermediate risk	High risk	N	CR1 with undetectable MRD	NA	MSD	213
P21	19	Female	Ph-negative B-ALL	*WT1::ABL, JAK2, EP300*	Intermediate risk	High risk	N	CR1 with undetectable MRD	Positive	MSD	161

*Ph* Philadelphia chromosome, *B-ALL* B-lineage acute lymphoblastic leukemia, *B-LBL* B-lineage acute lymphoblastic lymphoma, *AA* aplastic anemia, *CML* chronic myelocytic leukemia, *R-DRI* Refined Disease Risk Index, *N* no, *Y* yes, *allo-HSCT* allogeneic hematopoietic stem cell transplantation, *CR1* first complete remission, *MRD* measurable residual disease detected with multiparameter flow cytometry, *CR2* second complete remission, *NR* no remission, *NA* not applicable, *MUD* matched unrelated donor, *MSD* matched sibling donor, *HID* haploidentical donor.

^a^Acute Lymphoblastic Leukemia, Version 4.2023, NCCN Clinical Practice Guidelines in Oncology.

The median time from transplantation to the start of blinatumomab therapy was 102 (range: 42–227) days. Patients completed a median of 2 (range: 1–6) courses (1 cycle: *n* = 10, 2 cycles: *n* = 6, 3 cycles: *n* = 2, ≥ 4 cycles: *n* = 3). The median interval between cycles of blinatumomab was 82 (range: 31–202) days. A total of 42 courses of blinatumomab were administered, with one treatment course interrupted due to COVID-19 infection. Reasons for discontinuation of treatment are described in Supplementary Table 3. Details regarding blinatumomab treatment are shown in Supplementary Table 4. One Ph-positive B-ALL patient received olverembatinib plus with blinatumomab as maintenance therapy. The treatment schedule for each patient is illustrated in Supplementary Fig. 1.

The most frequently AEs observed were cytopenias (Supplementary Table 5). Infections, including bloodstream and respiratory infections, were observed in 8 patients (38.1%). One patient (P5) experienced Epstein–Barr virus reactivation 10 days after completing the previous cycle of blinatumomab. Grade I cytokine release syndrome (CRS) occurred in three patients but was resolved with symptomatic treatment. No neurologic toxicities were reported.

Prior to maintenance therapy, 11 patients had discontinued their courses of immunosuppressants, while the remaining 10 patients were taking calcineurin inhibitors during the first maintenance cycle. The cumulative incidences of grades I-II and III-IV aGvHD were 28.6% (*n* = 6) and 4.8% (*n* = 1), respectively, and cases occurred at a median of 31 (range:13–52) days after the initiation of blinatumomab. Five patients were diagnosed with cGvHD (mild/moderate cGvHD: *n* = 4; severe cGvHD: *n* = 1, Supplementary Table 6) at a median of 78 (35–143) days after the initiation of blinatumomab maintenance. Following anti-GvHD therapies, the complete response rate was 100% in patients with aGvHD and 80% in patients with cGvHD.

There was a median follow-up time of 325 (range: 156–775) days for all patients, and 18 patients (85.7%) were alive at the end of the study. Causes of death included relapsed/progressive disease (*n* = 1) and NRM (transplant-associated thrombotic microangiopathy: *n* = 1; infection: *n* = 1). The relapse rate was 6.3%, representing a refractory/relapsed B-ALL patient (P13) who was in second CR with detectable MRD at allo-HSCT. No NRM events were attributable to blinatumomab. The clinical outcomes of all patients are displayed in Supplementary Table 7. The 1-year OS, EFS, and GRFS for the entire cohort were 81.6% (95% CI 64.7–100%), 82.1% (95% CI 65.6–100%) and 82.5% (95% CI 66.2–100%), respectively (Fig. 1). Of the 15 patients in their first CR with undetectable MRD at allo-HSCT, one died from NRM (infection), and no relapses were reported. The 1-year OS, EFS, and GRFS for these patients were 92.9% (95% CI 80.3–100%), 92.9% (95% CI 80.3–100%), and 93.3% (95% CI 81.5–100%), respectively. For non-refractory/relapsed B-LBL/ALL patients, the one-year OS and EFS were 100% and 100%, respectively.

**Fig. 1:**
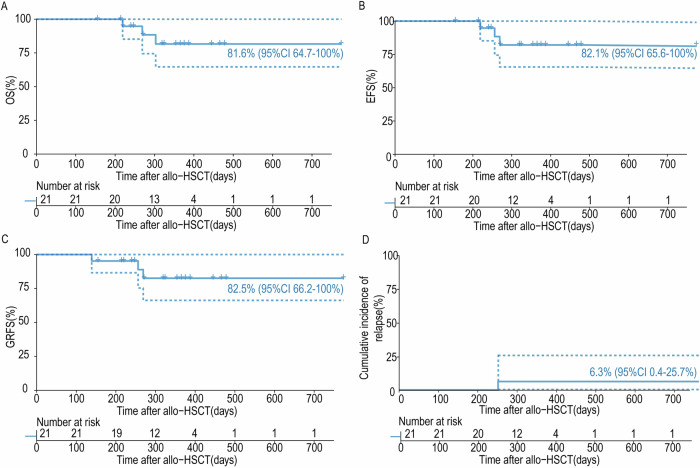
Survival outcomes of 21 patients on blinatumomab maintenance post allo-HSCT. Survival curves plotted against number of days after allo-HSCT for (**A**) overall survival (OS), (**B**) event-free survival (EFS), (**C**) GvHD-free and relapse-free survival (GRFS), (**D**) relapse. The data is presented as percentage with 95%CI.

Stein et al. [12] reported that blinatumomab produced a 45% CR rate for B-ALL patients who relapsed following allo-HSCT. However, despite this salvage treatment, approximately 80% of patients experienced relapse within one year, leading to a poor long-term OS rate of 36% at 1 year and 18% at 3 years. The use of prophylactic blinatumomab for high-risk patients who have achieved hematological CR after allo-HSCT is expected to enhance the anti-leukemic effect of engrafted donor-derived CD3-positive T cells. In a phase 2b trial, the MD Anderson group investigated the efficacy and safety of blinatumomab following allo-HSCT in 21 patients. They reported that the 81% of patients (*n* = 17) who received grafts from HLA-matched donors had 1-year OS and PFS rates of 85% and 71%, respectively [13]. In our study, all patients achieved CR with undetectable MRD before the initiation of blinatumomab treatment; additionally, 81% of the patients (*n* = 17) received grafts from haploidentical donors, distinguishing our study from the experience of the MD Anderson group. A phase Ib/II trial that enrolled high-risk CD19-positive B-ALL and non-Hodgkin lymphoma patients who had attained MRD-negative CR after allo-HSCT recorded a 3-year RFS of 73% [14]. In the Campus ALL study, blinatumomab led to a transient redistribution of effector T-cell subsets and Treg cells, along with a persistent increase in cytotoxic NK cells in the peripheral blood [15].

A lower leukemia burden is a positive indicator for effective immunotherapy. All alive patients in their first CR with undetectable MRD at the time of allo-HSCT achieved sustained MRD remission after receiving blinatumomab maintenance (P3 died of NRM). Nine patients were in refractory/relapsed B-ALL/LBL before allo-HSCT, and six patients achieved sustained MRD remission after allo-HSCT and blinatumomab maintenance (P13 died of relapse, and P3 and P15 died of NRM).

Concerns regarding the potential risks of blinatumomab-associated immune-mediated toxicities following allo-HSCT have not been adequately addressed in the literature. These include the exacerbation of GvHD, delayed engraftment, and graft failure or rejection. In the present study, no increase in NRM incidences was observed within our cohort. CRS and encephalopathy syndrome were identified as AEs of interest, and the incidences of grade I CRS and grade III-IV aGvHD were 14.3% and 4.8%, respectively. Blinatumomab was well tolerated by patients, and there was a low incidence of myelosuppression. Notably, three patients in our cohort were aged ≤ 18 years, and no additional AEs were observed in these patients.

The findings of the present study suggest that blinatumomab maintenance therapy following allo-HSCT is feasible, efficacious, and safe. It is important to note that our study had certain limitations, including its retrospective design, small cohort size, and relatively short follow-up period. Therefore, further prospective, randomized studies are needed to identify patients who would benefit most from blinatumomab maintenance therapy after allo-HSCT.

### Supplementary information

Supplemental Materials

## Data Availability

The datasets generated and/or analyzed during the current study are available from the corresponding author on reasonable request.

## References

[1] Acute Lymphoblastic Leukemia, Version 4.2023, NCCN Clinical Practice Guidelines in Oncology. National Comprehensive Cancer Network. http://www.nccn.org.

[2] Piemontese S, Boumendil A, Labopin M, Schmid C, Ciceri F, Arcese W (2019). Leukemia relapse following unmanipulated haploidentical transplantation: a risk factor analysis on behalf of the ALWP of the EBMT. J Hematol Oncol.

[3] Wang Z, Fan Z, Wu Z, Xuan L, Li X, Tang B (2024). PASS-ALL study of paediatric-inspired versus adult chemotherapy regimens on survival of high-risk Philadelphia-negative B-cell acute lymphoblastic leukaemia with allogeneic haematopoietic stem cell transplantation. Br J Haematol.

[4] Jabbour E, Short NJ, Jain N, Haddad FG, Welch MA, Ravandi F (2023). The evolution of acute lymphoblastic leukemia research and therapy at MD Anderson over four decades. J Hematol Oncol.

[5] Liu H, Xuan L, Lin R, Deng L, Fan Z, Nie D (2021). A new pre-emptive TKIs strategy for preventing relapse based on BCR/ABL monitoring for Ph+ALL undergoing allo-HCT: a prospective clinical cohort study. Leukemia.

[6] Fan J, Lu R, Zhu J, Guo X, Wan D, Xie X (2023). Effects of post-transplant maintenance therapy with decitabine prophylaxis on the relapse for acute lymphoblastic leukemia. Bone Marrow Transpl.

[7] Lu W, Lyu H, Xiao X, Bai X, Zhang M, Wang J, et al. Prophylactic donor-derived CD19 CAR-T cell infusion for preventing relapse in high-risk B-ALL after allogeneic hematopoietic stem cell transplantation. Leukemia. 2024. 10.1038/s41375-024-02251-5. 10.1038/s41375-024-02251-5 PMC11147756 38632315

[8] Metheny LL, Sobecks R, Cho C, Fu P, Margevicius S, Wang J (2024). A multicenter study of posttransplantation low-dose inotuzumab ozogamicin to prevent relapse of acute lymphoblastic leukemia. Blood Adv.

[9] Jabbour E, Zugmaier G, Agrawal V, Martínez-Sánchez P, Rifón Roca JJ, Cassaday RD (2024). Single agent subcutaneous blinatumomab for advanced acute lymphoblastic leukemia. Am J Hematol.

[10] Kantarjian H, Stein A, Gökbuget N, Fielding AK, Schuh AC, Ribera JM (2017). Blinatumomab versus Chemotherapy for Advanced Acute Lymphoblastic Leukemia. N. Engl J Med.

[11] Gökbuget N, Dombret H, Bonifacio M, Reichle A, Graux C, Faul C (2018). Blinatumomab for minimal residual disease in adults with B-cell precursor acute lymphoblastic leukemia. Blood.

[12] Stein AS, Kantarjian H, Gökbuget N, Bargou R, Litzow MR, Rambaldi A (2019). Blinatumomab for Acute Lymphoblastic Leukemia Relapse after Allogeneic Hematopoietic Stem Cell Transplantation. Biol Blood Marrow Transpl.

[13] Gaballa MR, Banerjee P, Milton DR, Jiang X, Ganesh C, Khazal S (2022). Blinatumomab maintenance after allogeneic hematopoietic cell transplantation for B-lineage acute lymphoblastic leukemia. Blood.

[14] Webster JA, Jones RJ, Blackford A, Shedeck A, Ambinder RF, Swinnen LJ (2023). A Phase IB/II Study of Blinatumomab in Patients with B-Cell Acute Lymphoblastic Leukemia (ALL) and B-Cell Non-Hodgkin Lymphoma (NHL) As Post-Allogeneic Blood or Marrow Transplant (alloBMT) Remission Maintenance. Blood.

[15] Ocadlikova D, Lussana F, Fracchiolla N, Bonifacio M, Santoro L, Delia M (2023). Blinatumomab differentially modulates peripheral blood and bone marrow immune cell repertoire: A Campus ALL study. Br J Haematol.

