# Supplementary material for: Efficacy of blinatumomab as maintenance therapy for B-lineage acute lymphoblastic leukemia/lymphoma following allogeneic hematopoietic cell transplantation
Source: Blood Cancer J. 2024 Jul 8;14(1):109. doi: 10.1038/s41408-024-01092-w (PMC11231304; doi:10.1038/s41408-024-01092-w)
Supplement: Supplementary file 1 — Supplemental Materials [file 41408_2024_1092_MOESM1_ESM.docx]

**Supplemental Appendix**

**Efficacy of blinatumomab as maintenance therapy for B-lineage acute lymphoblastic leukemia/lymphoma following allogeneic hematopoietic cell transplantation**

**Contents**

**Supplementary Methods**

MRD detection 2

Transplantation protocol and aGvHD prophylaxis 2

Definitions of endpoints 2

**Supplementary Figure**

Supplementary Figure 1 Swimmer plot of treatment schedule and outcomes for individual patients 3

**Supplementary Tables**

Supplementary Table 1 Baseline characteristics of 21 patients with blinatumomab maintenance post-allo-HSCT 4-5

Supplementary Table 2 Treatment history before allo-HSCT 6

Supplementary Table 3 Reasons for blinatumomab discontinuation 7

Supplementary Table 4 Detailed information on blinatumomab maintenance 8-9

Supplementary Table 5 Adverse events of blinatumomab maintenance 10

Supplementary Table 6 Acute and chronic GvHD after blinatumomab maintenance 11

Supplementary Table 7 Clinical outcomes of blinatumomab maintenance 12

**Reference** 13

**Supplementary Methods**

**MRD detection**

Measurable residual disease (MRD) was assessed using multiparameter flow cytometry (MFC), with 0.01% as the threshold to distinguish MRD positivity [1]. The recurrent fusion gene was identified with RNA-seq and validated with quantitative (q)PCR. Sanger sequencing was used to detect concurrent mutations.

**Transplantation protocol and aGvHD prophylaxis**

The preconditioning regimen included cytarabine, busulfan, cyclophosphamide, semustine, and anti-thymocyte globulin [2]. The protocols for graft-versus-host disease (GvHD) and infection prophylaxis were as reported previously [3-4].

**Definitions of endpoints**

Overall survival was measured from the time of allo-HSCT to death or recorded as censored on the last follow-up. Event-free survival was defined as the interval between remission and MFC-MRD positivity, relapse, or death, or censored at the last follow-up. GvHD-free and relapse-free survival were calculated from allo-HSCT to death, as grades III-IV aGvHD, relapse, or censored on the last follow-up. Blinatumomab-associated events (i.e., infection, GvHD, virus reactivation, cytopenia) were defined as those that developed within 3 months after blinatumomab therapy or according to the treating physician’s opinion. Acute GvHD and chronic GvHD (cGvHD) were retrospectively defined and graded based on the Mount Sinai Acute GvHD International Consortium criteria [5] and NIH 2014 criteria [6]. The evaluation of adverse event grade was based on the National Cancer Institute’s Common Terminology Criteria for Adverse Events v5.0.

**Supplementary Figure 1. Swimmer plot of treatment schedule and outcomes in individual patients**

**
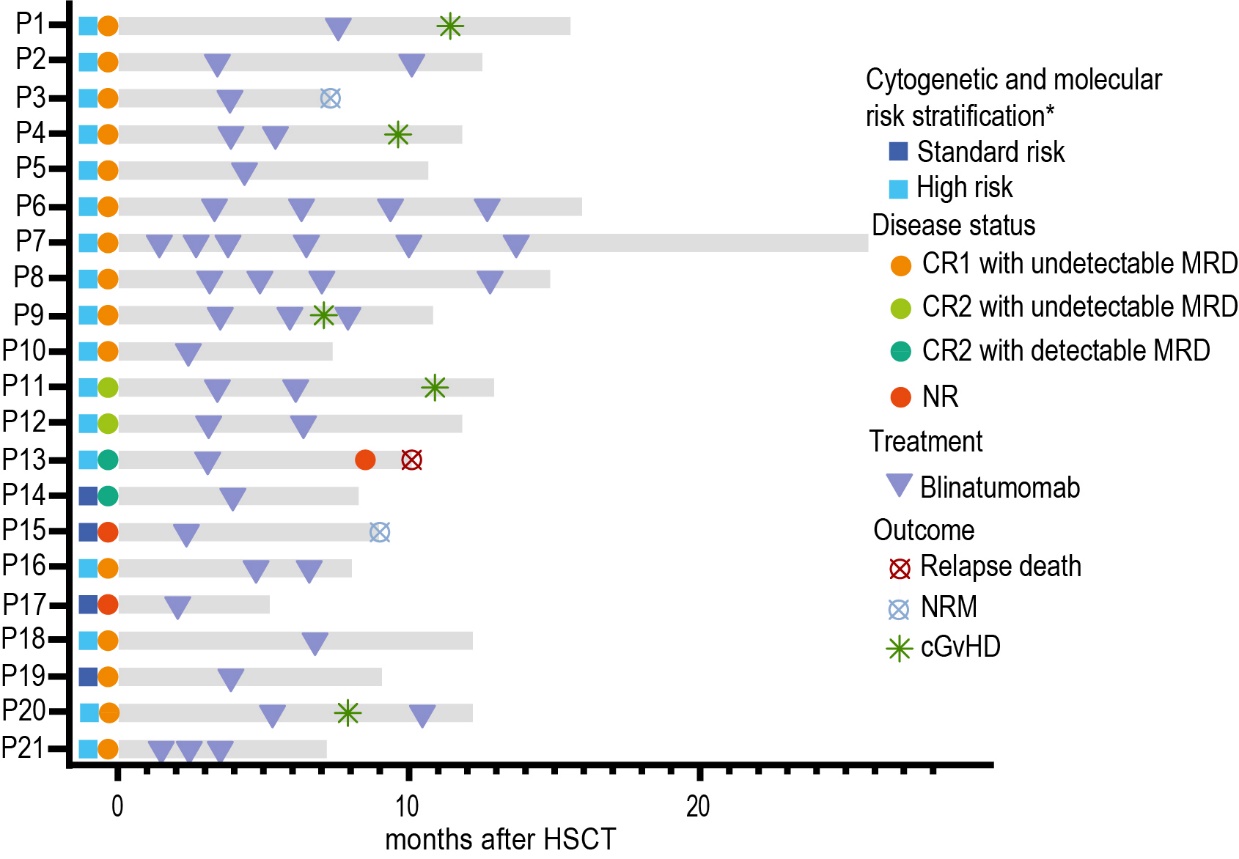
**

*Acute Lymphoblastic Leukemia, Version 4.2023, NCCN Clinical Practice Guidelines in Oncology

**Supplementary Table 1. Baseline characteristics of 21 patients with blinatumomab maintenance post-allo-HSCT**

| **Variable** | **Total** |
| --- | --- |
|  | **(n = 21)** |
| Age at allo-HSCT (median, range) | 29 (14-60) |
| Gender (*n*, %) |  |
| Male | 13 (61.9) |
| Female | 8 (38.1) |
| Days from diagnosis/first relapse to HSCT (median, range) | 162 (55-2039) |
| Disease status at HSCT (*n*, %) |  |
| First CR with undetectable MFC-MRD | 15 (71.5) |
| Second CR with undetectable MFC-MRD | 2 (9.5) |
| Second CR with detectable MFC-MRD | 2 (9.5) |
| NR | 2 (9.5) |
| R-DRI (*n*, %) |  |
| Intermediate risk | 15 (71.4) |
| High risk | 6 (28.6) |
| Cytogenetic and molecular prognostic risk stratification* (*n*, %) |  |
| Standard risk | 4 (19.0) |
| High risk | 17 (81.0) |
| Refractory/relapsed B-ALL/LBL* (*n*, %) |  |
| Refractory/relapsed | 9 (42.9) |
| Non-refractory/relapsed | 12 (57.1) |
| Donor type (*n*, %) |  |
| HID | 17 (81.0) |
| MUD | 1 (4.8) |
| MSD | 3 (14.2) |
| GvHD prophylaxis (*n*, %) |  |
| ATG-based | 12 (57.1) |
| PTCy-based | 8 (38.1) |
| CSA+MTX+MMF | 1 (4.8) |
| CD34 (×10^6^/kg) (median, range) | 7.22 (2.3-12.9) |
| MNC (×10^8^/kg) (median, range) | 10.37 (5-20.7) |

Abbreviations: allo-HSCT: allogeneic hematopoietic stem cell transplantation; CR: complete remission; MFC-MRD: measurable residual disease detected with multiparameter flow cytometry; NR: no remission; R-DRI: Refined Disease Risk Index; B-ALL/LBL: B-lineage acute lymphoblastic leukemia/lymphoma; HID: haploidentical donor; MUD: matched unrelated donor; MSD: matched sibling donor; GvHD: graft-versus-host disease; ATG: anti-lymphocyte globulin; PTCy: posttransplant cyclophosphamide; CSA: cyclosporin A; MTX: methotrexate; MMF: mycophenolate mofetil; MNC: mononucleated cells.

*Acute Lymphoblastic Leukemia, Version 4.2023, NCCN Clinical Practice Guidelines in Oncology

**Supplementary Table 2: Treatment history before allo-HSCT**

| **Patient** | **Treatment history** |
| --- | --- |
| P1 | Chemo induction🞨1-Chemo consolidation🞨1-Blina consolidation🞨1 |
| P2 | Chemo induction🞨1- Chemo consolidation🞨1-Blina+Chemo consolidation🞨1 |
| P3 | Chemo induction🞨1-Blina induction🞨1-Blina consolidation🞨1 |
| P4 | Chemo induction🞨1-Chemo consolidation🞨1-Blina consolidation🞨1 |
| P5 | Chemo induction🞨1-Chemo consolidation🞨4-Blina consolidation🞨3 |
| P6 | Chemo induction🞨1-Blina induction🞨1-Blina consolidation🞨1 |
| P7 | Chemo induction🞨1-Chemo consolidation🞨2-Blina consolidation🞨2 |
| P8 | Chemo induction🞨1-Blina+Chemo consolidation🞨2 |
| P9 | Chemo induction🞨1-Blina induction🞨1-Blina consolidation🞨1 |
| P10 | Chemo induction🞨2 |
| P11 | Chemo induction🞨1-Chemo consolidation🞨2-allo-HSCT-CAR-T-relapse  Blina re-induction🞨1 |
| P12 | Chemo induction🞨1-Chemo consolidation🞨3-allo-HSCT-relapse  Blina+Chemo re-induction🞨1-Chemo consolidation🞨1 |
| P13 | Chemo induction🞨1-Chemo consolidation🞨10-allo-HSCT-relapse  Chemo re-induction🞨1-Blina consolidation🞨1 |
| P14 | Chemo induction🞨1-Chemo consolidation🞨2-relapse-Chemo re-induction🞨1 |
| P15 | Chemo induction🞨4-auto-HSCT-relapse-Chemo re-induction🞨1-Blina consolidation🞨1-NR |
| P16 | Flumatinib -Chemo induction🞨1+ Olverembatinib -Blina induction🞨1+ Olverembatinib |
| P17 | Chemo induction🞨1-Chemo consolidation🞨6- extramedullary relapse (testicles)  Chemo re-induction🞨1-Radiotherapy-Chemo re-induction🞨2- extramedullary relapse (CNS)  Chemo re-induction🞨1- intrathecal chemotherapy🞨5-NR (CNS+intramedullary relapse) |
| P18 | Chemo induction🞨1-Chemo consolidation🞨3 |
| P19 | Chemo induction🞨1 |
| P20 | Chemo induction🞨5 |
| P21 | Chemo induction🞨1-Chemo consolidation🞨2 |

Abbreviations: allo-HSCT: allogeneic hematopoietic stem cell transplantation; Chemo: chemotherapy; Blina: blinatumomab; auto-HSCT: autologous stem cell transplantation; CAR-T: chimeric antigen receptor (CAR)-T cell therapy; TKI: tyrosine kinase inhibitor; CNS: central nervous system; NR: no remission.

**Supplementary Table 3. Reasons for blinatumomab discontinuation**

| **Cause** | **n** |
| --- | --- |
| Medical insurance | 6 |
| Infection secondary to blinatumomab | 3 |
| Chronic graft-versus-host disease | 5 |
| Within three months from last blinatumomab cycle upon last follow-up | 4 |

**Supplementary Table 4. Detailed information on blinatumomab maintenance**

| **Patient** | **Molecular alteration**  **before HSCT**† | **Molecular alteration**  **before maintenance^#^** | **No. courses** | **1^st^*** | **Dose** | **2^nd^*** | **Dose** | **3^rd^*** | **Dose** | **4^th^*** | **Dose** | **5^th^*** | **Dose** | **6^th^*** | **Dose** |
| --- | --- | --- | --- | --- | --- | --- | --- | --- | --- | --- | --- | --- | --- | --- | --- |
| P1 | Negative | Negative | 1 | 227 | 9 µg d1-7, 28 µg d8-14 |  |  |  |  |  |  |  |  |  |  |
| P2 | Negative | Negative | 2 | 102 | 28 µg d1-4, d47-56 | 304 | 9 µg d1-7, 28 µg d8-14 |  |  |  |  |  |  |  |  |
| P3 | Negative | Negative | 1 | 115 | 28 µg d1-8, d15-20 |  |  |  |  |  |  |  |  |  |  |
| P4 | Negative | Negative | 2 | 116 | 28 µg d1-14 | 216 | 28 µg d1-14 |  |  |  |  |  |  |  |  |
| P5 | NA | NA | 1 | 130 | 9 µg d1-3, 28 µg d4-14 |  |  |  |  |  |  |  |  |  |  |
| P6 | Negative | Negative | 4 | 99 | 9 µg d1-3, 28 µg d4-14 | 189 | 9 µg d1-3, 28 µg d4-14 | 281 | 9 µg d1-3, 28 µg d4-14 | 382 | 9 µg d1-3, 28 µg d4-14 |  |  |  |  |
| P7 | Negative | Negative | 6 | 42 | 28 µg d1-14 | 80 | 28 µg d1-14 | 113 | 28 µg d1-14 | 194 | 28 µg d1-14 | 301 | 28 µg d1-14 | 410 | 28 µg d1-14 |
| P8 | Positive | Negative | 4 | 94 | 9 µg d1-3, 28 µg d4-14 | 146 | 9 µg d1-3, 28 µg d4-14 | 210 | 9 µg d1-3, 28 µg d4-14 | 385 | 9 µg d1-3, 28 µg d4-14 |  |  |  |  |
| P9 | Negative | Negative | 3 | 105 | 9 µg d1-3, 28 µg d4-14 | 177 | 28 µg d1-14 | 238 | 28 µg d1-14 |  |  |  |  |  |  |
| P10 | Positive | Negative | 1 | 71 | 9 µg d1-3, 28 µg d4-14 |  |  |  |  |  |  |  |  |  |  |
| P11 | Negative | Negative | 2 | 102 | 9 µg d1-3, 28 µg d4-14 | 184 | 28 µg d1-14 |  |  |  |  |  |  |  |  |
| P12 | Negative | Negative | 2 | 93 | 9 µg d1-3, 28 µg d4-14 | 191 | 9 µg d1-3, 28 µg d4-14 |  |  |  |  |  |  |  |  |
| P13 | Positive | Negative | 1 | 92 | 9 µg d1-3, 28 µg d4-14 |  |  |  |  |  |  |  |  |  |  |
| P14 | NA | NA | 1 | 118 | 9 µg d1-3, 28 µg d4-14 |  |  |  |  |  |  |  |  |  |  |
| P15 | Positive | Negative | 1 | 70 | 28 µg d1-14 |  |  |  |  |  |  |  |  |  |  |
| P16 | Negative | Negative | 2 | 142 | 9 µg d1-3, 28 µg d4-14 | 197 | 9 µg d1-3, 28 µg d4-14 |  |  |  |  |  |  |  |  |
| P17 | NA | NA | 1 | 61 | 9 µg d1-14 |  |  |  |  |  |  |  |  |  |  |
| P18 | NA | NA | 1 | 203 | 9 µg d1-3, 28 µg d4-14 |  |  |  |  |  |  |  |  |  |  |
| P19 | NA | NA | 1 | 116 | 9 µg d1-3, 28 µg d4-14 |  |  |  |  |  |  |  |  |  |  |
| P20 | NA | NA | 2 | 159 | 28 µg d1-7 | 314 | 28 µg d1-7 |  |  |  |  |  |  |  |  |
| P21 | Positive | Positive | 3 | 44 | 28 µg d1-7 | 73 | 28 µg d1-7 | 104 | 28 µg d1-7 |  |  |  |  |  |  |

Note: ^†^Before allo-HSCT; ^#^Before blinatumomab maintenance; *Days from allo-HSCT to blinatumomab maintenance.

**Supplementary Table 5 Adverse events during blinatumomab maintenance**

| **Toxicity** | **Maximum grade** | | | |
| --- | --- | --- | --- | --- |
|  | **1 (n, %)** | **2 (n, %)** | **3 (n, %)** | **4 (n, %)** |
| Anemia | 0 (0) | 2 (9.5) | 0 (0) | 0 (0) |
| Neutropenia | 0 (0) | 2 (9.5) | 6 (28.6) | 5 (23.8) |
| Thrombopenia | 2 (9.5) | 2 (9.5) | 4 (19.0) | 2 (9.5) |
| Infections | 0 (0) | 3 (14.2) | 4 (19.0) | 1 (4.7) |
| CRS | 3 (14.2) | 0 (0) | 0 (0) | 0 (0) |
| Elevated liver enzymes | 2 (9.5) | 1 (4.7) | 0 (0) | 0 (0) |
| Hypogeusia | 1 (4.7) | 0 (0) | 0 (0) | 0 (0) |

Abbreviations: CRS**:** cytokine release syndrome

**Supplementary Table 6. Acute and chronic GvHD after blinatumomab maintenance**

| **Patient** | **aGvHD*** | **Organ/stage** | **Interval since previous cycle (d)** | **cGvHD^#^** | **Organ/stage** | **Interval since previous cycle (d)** |
| --- | --- | --- | --- | --- | --- | --- |
| P1 |  |  |  | Mild | Skin score 1 | 116 |
| P2 | Grade I | Skin stage 1 | 32 |  |  |  |
| P3 | Grade III | Skin stage 1, Gut stage 3 | 30 |  |  |  |
| P4 |  |  |  | Mild | Eye score 1, Joint score 1 | 73 |
| P5 | Grade II | Skin stage 3 | 13 |  |  |  |
| P9 | Grade I | Skin stage 1 | 40 | Mild | Skin score 1 | 35 |
| P10 | Grade I | Skin stage 1 | 52 |  |  |  |
| P11 | Grade I | Skin stage 1 | 31 | Moderate | Lung score 1 | 143 |
| P17 | Grade I | Skin stage 1 | 20 |  |  |  |
| P20 |  |  |  | Severe | Liver score 3 | 78 |

Abbreviations: GvHD: graft-versus-host-disease; aGvHD: acute GvHD; cGvHD: chronic GvHD; d: days

*Based on Mount Sinai Acute GvHD International Consortium criteria^[5]^

^#^Based on NIH 2014 criteria^[6]^

**Supplementary Table 7.** **Clinical outcomes with blinatumomab maintenance**

| **Outcome** | **Diagnosis (*n*,%)** | |  | **MFC-MRD at allo-HSCT (*n*, %)** | |  | **Cytogenetic and molecular prognostic risk stratification* (*n*, %)** | |
| --- | --- | --- | --- | --- | --- | --- | --- | --- |
|  | **De novo** | **relapsed/refractory** |  | **Negative** | **Positive** |  | **Standard risk** | **High risk** |
| 1-year event-free survival | 12(100) | 6(66.7) |  | 16 (76.2) | 2 (50) |  | 3 (75) | 15 (88.2) |
| 1-year relapse-related mortality | 0(0) | 1(11.1) |  | 0 (0) | 1 (25) |  | 0 (0) | 1 (5.9) |
| 1-year NRM | 0(0) | 2(22.2) |  | 1 (4.8) | 1 (25) |  | 1 (25) | 1 (5.9) |

Abbreviations: MFC-MRD: measurable residual disease detected with multiparameter flow cytometry; allo-HSCT: allogeneic hematopoietic stem cell transplantation; NRM: non-relapse mortality; R-DRI: Refined Disease Risk Index.

* Acute Lymphoblastic Leukemia, Version 4.2023, NCCN Clinical Practice Guidelines in Oncology

References

1. Kotrova M, Koopmann J, Trautmann H, Alakel N, Beck J, Nachtkamp K, et al. Prognostic value of low-level MRD in adult acute lymphoblastic leukemia detected by low- and high-throughput methods. Blood Adv. 2022; 6(10): 3006-10.

2. Wang Y, Liu QF, Xu LP, Liu KY, Zhang XH, Ma X, et al. Haploidentical vs identical-sibling transplant for AML in remission: a multicenter, prospective study. Blood. 2015; 125(25): 3956-62.

3. Wang Y, Liu QF, Lin R, Yang T, Xu YJ, Mo XD, et al. Optimizing antithymocyte globulin dosing in haploidentical hematopoietic cell transplantation: long-term follow-up of a multicenter, randomized controlled trial. Science Bulletin. 2021; 66(24): 2498-505.

4. Shen MZ, Hong SD, Lou R, Chen RZ, Zhang XH, Xu LP, et al. A comprehensive model to predict severe acute graft-versus-host disease in acute leukemia patients after haploidentical hematopoietic stem cell transplantation. Experimental hematology & oncology. 2022;11(1):25.

5. Harris AC, Young R, Devine S, Hogan WJ, Ayuk F, Bunworasate U, et al. International, Multicenter Standardization of Acute Graft-versus-Host Disease Clinical Data Collection: A Report from the Mount Sinai Acute GVHD International Consortium. Biology of Blood and Marrow Transplantation. 2016;22(1):4-10.

6. Jagasia MH, Greinix HT, Arora M, Williams KM, Wolff D, Cowen EW, et al. National Institutes of Health Consensus Development Project on Criteria for Clinical Trials in Chronic Graft-versus-Host Disease: I. The 2014 Diagnosis and Staging Working Group Report. Biology of Blood and Marrow Transplantation. 2015;21(3):389-401.e1.
